# Waterborne Graphene- and Nanocellulose-Based Inks for Functional Conductive Films and 3D Structures

**DOI:** 10.3390/nano11061435

**Published:** 2021-05-29

**Authors:** Jose M. González-Domínguez, Alejandro Baigorri, Miguel Á. Álvarez-Sánchez, Eduardo Colom, Belén Villacampa, Alejandro Ansón-Casaos, Enrique García-Bordejé, Ana M. Benito, Wolfgang K. Maser

**Affiliations:** 1 Instituto de Carboquímica ICB-CSIC, C/Miguel Luesma Castán 4, 50018 Zaragoza, Spain; baigorri1994@gmail.com (A.B.); maalvarez@icb.csic.es (M.Á.Á.-S.); ecolom@icb.csic.es (E.C.); alanson@icb.csic.es (A.A.-C.); jegarcia@icb.csic.es (E.G.-B.); abenito@icb.csic.es (A.M.B.); wmaser@icb.csic.es (W.K.M.); 2 Department of Condensed Matter Physics, ICMA-CSIC, University of Zaragoza, 50009 Zaragoza, Spain; bvillaca@unizar.es

**Keywords:** graphene, carbon nanotubes, nanocellulose, conductive inks, liquid-phase processing, film fabrication, sustainability, metal-free electrodes

## Abstract

In the vast field of conductive inks, graphene-based nanomaterials, including chemical derivatives such as graphene oxide as well as carbon nanotubes, offer important advantages as per their excellent physical properties. However, inks filled with carbon nanostructures are usually based on toxic and contaminating organic solvents or surfactants, posing serious health and environmental risks. Water is the most desirable medium for any envisioned application, thus, in this context, nanocellulose, an emerging nanomaterial, enables the dispersion of carbon nanomaterials in aqueous media within a sustainable and environmentally friendly scenario. In this work, we present the development of water-based inks made of a ternary system (graphene oxide, carbon nanotubes and nanocellulose) employing an autoclave method. Upon controlling the experimental variables, low-viscosity inks, high-viscosity pastes or self-standing hydrogels can be obtained in a tailored way. The resulting inks and pastes are further processed by spray- or rod-coating technologies into conductive films, and the hydrogels can be turned into aerogels by freeze-drying. The film properties, with respect to electrical surface resistance, surface morphology and robustness, present favorable opportunities as metal-free conductive layers in liquid-phase processed electronic device structures.

## 1. Introduction

Liquid-phase processing (LPP) is currently the preferred route for building up advanced layered film devices such as environmental, physical or biological sensors, logic circuits, radiofrequency transmitters or screens [1]. The cornerstone of this field is to integrate high-performance electronic materials into functional systems in a low-cost configuration, with high performance and ease of manufacturing [2]. Therefore, the wide diversity of deposition methods in the liquid phase are very suitable for this purpose, and able to provide high-resolution patterns through liquid inks in a fully scalable manner [3,4]. Inks are complex mixtures of certain components and additives within a liquid medium (generally an organic and high boiling point solvent) whose concentrations and chemical nature determine their physical properties, namely rheology, surface tension and conductivity. The development of inks based on electrically conductive species is therefore critical to progress in this field of work [4,5].

Conductive nanomaterials have attracted considerable interest in this regard, as they offer excellent electronic properties and high compatibility with LPP [5]. Among the most studied nanostructures, those based upon carbon, such as carbon nanotubes (CNTs) and graphene derivatives, stand out. They possess extraordinary electrical, thermal and mechanical properties [5], as well as being extremely light and prone to biocompatibility. Recent efforts of the scientific community in the area of LPP of carbon nanostructures have focused on the development of inks, showing enormous potential in technological applications and some advantages like the abundance of the source material (graphite) [6]. Beyond the graphene materials obtained by direct graphite exfoliation [7], the scientific community has also successfully employed its related chemical derivatives, such as graphene oxide (GO) or reduced GO.

Traditionally, noble metals have been incorporated into conductive inks, at concentrations of ~60% of conductive metal to reach acceptable conductivity values. In addition to the high cost involved, their high concentration generates problems of chemical stability and reaction with other neighboring species such as air and solvents. Graphene-based nanostructures can solve this drawback due to their high chemical stability [3,8]. Moreover, conductive aqueous inks with metals can cause toxicity [9], while inks based on aqueous solutions of graphene have been tested in human skin cells, resulting in neither toxicity nor morphological changes at a cellular level [10]. Therefore, these inks may be safe if the rest of the additives are harmless. In this sense, GO, as a hydrophilic derivative of graphene, has shown excellent performance when processed from water-based dispersions into graphene-based conductive films [11]. Thus, to date, graphene material-based inks have demonstrated their superiority in a wide range of functionalities, such as flexible interconnections, electrodes, transparent conductors and supercapacitors [12]. Surface electrical resistance values in the range of ~100 Ω/□ are required for acting as electrode materials in organic or perovskite solar cell devices [13,14]. Thus, in order to establish versatility in the application of a certain conductive material by use of LPP, it is necessary to control the resistivity of the deposited layer. Furthermore, its roughness and compaction mainly depend on the deposition method, influenced in turn by the viscosity of the ink. The preparation of inks made of graphene-based nanomaterials is hence a challenging task, since several rheological properties of the ink (namely density, surface tension and viscosity) have a great impact [15].

As a matter of fact, high-viscosity inks (hereafter called pastes), with viscosities higher than 500 cP, would be suitable to form thicker layers (for instance, by screen printing techniques), while those with less viscosity would be more suitable for spray or inkjet deposition, allowing for a finer patterning and, in particular, for the case of spray coating technologies, the coverage of large areas. In general, these values can be adjusted by experimental parameters such as the concentration of the conductive additive or the dispersant, and also by the solvent choice. As mentioned earlier, typical liquid media in inks are non-volatile organic solvents. The most used ones for the production graphene-based liquid suspensions are *N*-methyl-2-pyrrolidone (NMP), *N*,*N*-dimethylformamide (DMF) and dimethylsulfoxide (DMSO), which are the ones that behave best as liquid-phase exfoliants [16]. These are very polluting solvents, with a boiling point of >170 °C, posing a serious risk of toxicity in humans. However, the use of water does not automatically solve these problems either, since it requires the incorporation of high concentrations of surfactants and other additives [9,17,18], indefinitely remaining in the ink, possibly leading to environmental and toxicity problems. Thus, new methods compatible with lower boiling point solvents (such as water or alcohols), together with non-toxic dispersants, are in demand in order to attain a truly environmentally friendly LPP, without raising the manufacturing cost or jeopardizing the overall electric/optoelectronic device performance once the deposition method takes place. Graphene-based nanostructures have promising potential, amongst currently known nanomaterials, to fulfill this purpose [19]. In particular, the GO derivatives have advantageous features for their use in conductive inks, such as affordable commercial availability, and the facile LPP due to the huge content of oxygen groups, in turn responsible for the higher interlayer spacing between planes. As stated by many authors, the GO LPP in a myriad of solvents and media does not need the addition of stabilizers, meaning a processing benefit [19]. For this reason, we have chosen GO as a target of interest for the preparation of aqueous inks. The only drawback is that GO inks require a subsequent reduction step to turn those inks into conductive material, be it by chemical, thermal or electrochemical means, usually entailing acceptable but poorer conductive properties than pristine graphene due to a larger number of structural defects [19]. Therefore, in the present work, the joint action of GO and CNTs was pursued to optimize the eventual conductive properties.

However, if one wishes to process carbon nanostructures from the liquid phase with greener approaches, avoiding toxic solvents or surfactants, a game-changing strategy is needed. In such a scenario, nanocrystalline cellulose (NCC) in particular acquires great relevance because of its sustainability. This nanomaterial is obtained from natural cellulose sources, by selective hydrolysis of the non-crystalline domains [20], resulting in fibrillar or needle-like nanostructures with widths and thicknesses around 3–20 nm and lengths of a few hundred nanometers. Due to its intrinsic chemical nature and the sustainability of the source material, nanocellulose may be considered an environmentally friendly nanomaterial. Despite the scarcity of scientific studies, there are already some examples showing the enormous potential that nanocellulose has as an aqueous dispersing agent of reduced GO and carbon nanotubes [21,22]. The structural diversity of NCC is defined by its crystalline allomorphs, among which types I and II stand out [22]. While type I NCC (exhibiting cellulose chains parallel to each other) is dominant in nature, type II is artificially synthesized, presenting polymer chains in antiparallel arrangements and typically requiring extreme caustic conditions or recrystallization processes for its synthesis. However, we have recently implemented a method to synthesize both NCC allomorphs by one-pot acid hydrolysis with sulfuric acid without any post-treatment step [22]. We also demonstrated the feasibility of dispersing CNTs in NCC, without any previous chemical modification on the NCC, leading to very stable aqueous colloids with proven bioactivity towards colon cancer (Caco-2) cells. In fact, the combination of carbon nanostructures with nanocellulose is an emerging trend, leading to useful hybrid nanomaterials with potential applications in biomedicine [23]. Beyond their biological response, nanocellulose paves the way towards new conductive inks based on carbon nanostructures, both dispersible in water and obeying green principles.

In this work, we disclose the development of carbon nanostructure-based low-viscosity inks and high-viscosity pastes able to be processed into films, by taking advantage of the impressive properties of NCC, standing out as a sustainable and green dispersing agent in water. We herein present a parametric study of ternary inks or pastes, or even self-standing hydrogels, by combining GO, CNTs and NCC and using a hydrothermal method in an autoclave. Further, conductive films were fabricated by different deposition techniques, and also hydrogel-derived porous materials, with the potential to become reference components as LPP electrodes and interfaces in electric/optoelectronic layered film device structures, such as batteries, supercapacitors, sensors and solar cells, among others.

## 2. Materials and Methods

### 2.1. Starting Materials and Reagents

The source of cellulose used in this work was microcrystalline cellulose (MCC) powder from cotton linters with an average particle size of 20 microns (Sigma-Aldrich, San Luis, MO, USA, ref 310697). Sulfuric acid (98%) for acid hydrolysis was purchased from Labkem (Barcelona, Spain). Ultrapure water with a conductivity of 0.055 μS/cm was obtained from a Siemens Ultraclear device (München, Germany) and used in every step of the present work. Multi-walled CNTs (MWCNTs) were acquired from NANOCYL^®^ (NC7000^TM^ variety), produced by a catalytic chemical vapor deposition method. Before use, MWCNTs underwent mild oxidation in liquid phase with HNO_3_ (1.5 M, 2 h under reflux) to render a more hydrophilic surface without compromising their structure [24]. GO came from a commercial aqueous dispersion (0.4 wt%, 4 mg/mL), purchased from Graphenea^®^ (San Sebastián, Spain). The characterization of mildly oxidized MWCNTs is presented in the Supplementary Materials (Figures S1 and S2), while the characterization of GO is provided by the manufacturer [25].

### 2.2. Synthesis of NCC (Types I and II)

As discussed in the Introduction, the synthesis of NCC was carried out according to a methodology developed in our laboratory [22]. In a typical experiment, 10 g of commercial MCC were added to 45 mL of ultrapure water inside a round-bottom flask, and dispersed with the aid of an ultrasonic bath for 10 min. The flask was then placed in an ice bath at 0 °C and 45 mL of 98% sulfuric acid were added dropwise, under constant magnetic stirring. Once added, the final concentration in the flask was 64% and the mixture was quite viscous, thus requiring vigorous and constant stirring to avoid locally high concentrations which could burn the cellulose. Right after the last droplet of acid was added, the flask was removed from the ice bath and transferred to a heating plate with magnetic stirring. At this point, the procedure varied according to the type of NCC to be obtained. For NCC type I, the reaction medium was heated for 10 min at 70 °C, while for type II it was heated for 5 h at 27 °C. Once the heating step was finished, the reaction medium was poured into ultrapure cold water, least ~10 times of the initial volume (90 mL) in a 1 L beaker and left overnight at 4 °C, in order to favor sedimentation. After decanting the supernatant liquid, a dispersion was left with a very acidic pH that was neutralized by dialysis. For this, the aqueous dispersion was inserted into a dialysis membrane (SpectraPor^®^, Spectrum Labs, regenerated cellulose, 6–8 kDa cutoff molecular weight) immersed in 5 L of ultrapure water. The dialysis water was changed periodically until neutral pH in the washing waters was achieved. Then, the dialyzed medium was centrifuged at ~9300 rcf, and the supernatant liquid was kept and subsequently freeze-dried, in order to use NCC as a fine and light powder. Full characterization of NCC can be found in reference [22].

### 2.3. Hydrothermal Treatment to Obtain Inks, Pastes and Hydrogels

Obtaining aqueous formulations able to be processed into films by different deposition techniques requires a certain control over their viscosity. This has been successfully attained through hydrothermal processes carried out in an autoclave. By heating a mixture of nanomaterials in water at a constant temperature (180 °C) in pressurized containers, with controlled times and pH, the chemical crosslinking of nanomaterials is favored [26]. In our case, this led to inks and pastes of different viscosities or even self-standing hydrogels. In general, what is herein termed as ink was of the order of 60 cP, while viscous pastes exhibited values of around 1500 cP.

We have undertaken a parametric study of different hydrothermal experiments, mixing variable amounts of NCC and CNTs and a fixed amount of GO, in order to ascertain the precise conditions under which inks, pastes or hydrogels form. All samples were brought to a final volume of 10 mL, and all samples were prepared at natural pH or alkalinized by the addition of aqueous ammonia (NH_4_OH), since basic pH plays a critical role by favoring the graphenic nanomaterials’ aggregation during hydrothermal treatment [26]. The experimental procedure followed was: in a flat-bottom quartz vial, a specific amount of NCC (yielding final concentrations of 2.5 mg/mL or 5 mg/mL) was weighed. Unless stated otherwise, NCC refers to its type I polymorph. Then, a certain amount of CNTs were added to the vial (to attain final concentrations of 0.1 mg/mL, 0.2 mg/mL, 0.5 mg/mL, 1 mg/mL or 2 mg/mL), enabling the analysis of the influence of the amount of CNTs on the conductivity and viscosity of the resulting composites. Further, a variable volume of ultrapure water (4.8 mL or 5.0 mL) was added depending on whether or not NH_4_OH (200 µL) was added, respectively. At this point, the vial was subjected to an ultrasound bath (45 kHz) for 3 min. After that, a semi-homogeneous dispersion could be observed inside the vial. Finally, a fixed volume (5.0 mL) of the aqueous dispersion of GO was added, resulting in a final GO concentration of 2 mg/mL. The resulting medium was again bath-sonicated for another 2 min, thus obtaining a homogeneous dispersion with a final volume of 10 mL.

**Figure 1:**
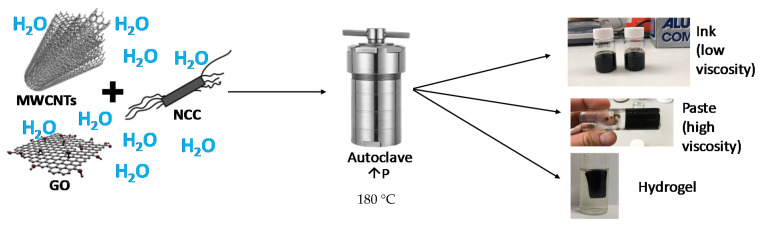
General scheme for the preparation of inks and pastes following the autoclave method.

The hydrothermal treatment consisted of placing the vials in a Teflon vessel, inserting it into a metallic autoclave, tightly closed, and then heating it in an oven. The autoclaves were placed in the oven after pre-heating it at 180 °C, with a heating ramp of 20 °C, and the treatment lasted for a specific duration. The studied times were mainly 60 min, 30 min and 15 min. After one of these times had elapsed, the autoclave was removed from the oven and allowed to cool down in ambient conditions to room temperature before taking the sample out of the autoclave. The time needed for the sample inside the autoclave to reach 180 °C was estimated to be compensated by the time needed for cooling down to a safe handling temperature, so the overall treatment times were consistent in all cases. Figure 1 shows a visual scheme of the process.

### 2.4. Film Preparation and Characterization

Once the inks (low-viscosity formulations) and pastes (high-viscosity formulations) were obtained by the procedures described above, the different possible deposition techniques were studied, in order to obtain the optimum conductive films. Spray-coating with an airbrush onto glass substrates was the chosen approach for the deposition of inks [27]. Films obtained from the viscous pastes were deposited by means of a rod-coating method using an agate rod, also over glass substrates. In both cases, the substrate was placed on a heating plate at 60 °C. For every sample, a total of 10 mL of the ink or paste was deposited in subsequent passages on the glass substrate, leading to films prepared under comparable conditions.

In order to measure the resistivity of the films, a Keithley 4200 unit was used, working in the range from −100 to 100 mV. An in-line 4-point probe configuration with equidistant probe separations of 2.24 mm was utilized, with controlled and homogeneous pressure over the conductive film. For the resistivity measurement, the geometry of the film and the distance between the electrodes were taken into account [18].

Morphology and microstructure of the deposited films were assessed through both optical and scanning electron (SEM) microscopies. Optical images were taken with a Zeiss AXIO microscope (Jena, Germany) with 20× and 50× objective lenses (N.A. = 0.4 and 0.7, respectively). SEM images were taken using Hitachi S3400N equipment (Tokyo, Japan), working in the secondary electron mode at a voltage of 15 kV and a distance of 5 mm.

Sample thickness as well as the roughness of the surface were evaluated by using a contact DektakXT Stylus Profiler (from Bruker, Billerica, MA, USA). The radius of the stylus used in the measurements was 2.5 µm. The height of the step of each deposited layer was measured in different areas along the layer edge. The depths of grooves made in the central part of the sample (reaching the substrate) were also determined. The layer thickness was obtained as the mean value of such measurements. The profile roughness was analyzed using Dektak analytical software (from Bruker, Jena, Germany).

### 2.5. Aerogel Preparation and Characterization

In order to obtain aerogels from hydrogels, a unidirectional freezing was applied before lyophilization. For that purpose, hydrogels were placed in an empty quartz vessel with a bottom platform made of metal which was immersed in liquid nitrogen. Once the hydrogel was fully frozen starting from the metal base, it was placed in a Telstar Cryodos freeze-drier, working at −49 °C and 0.3 mbar [28].

In order to measure the resistivity of the aerogels, the aforementioned Keithley unit was employed in a two-probe configuration. Aerogels were held between spring-loaded copper foils. The tungsten needle probes were brought into contact with the copper sheets, avoiding damage to the aerogels. The resistivities of the aerogels were calculated, taking into account the geometry, given by the distance between the electrodes and the specimen diameter, thus assuming a perfectly cylindrical shape.

## 3. Results and Discussion

### 3.1. Hydrothermal Development of Inks, Pastes and Hydrogels: Unraveling Critical Parameters

**Figure 2:**
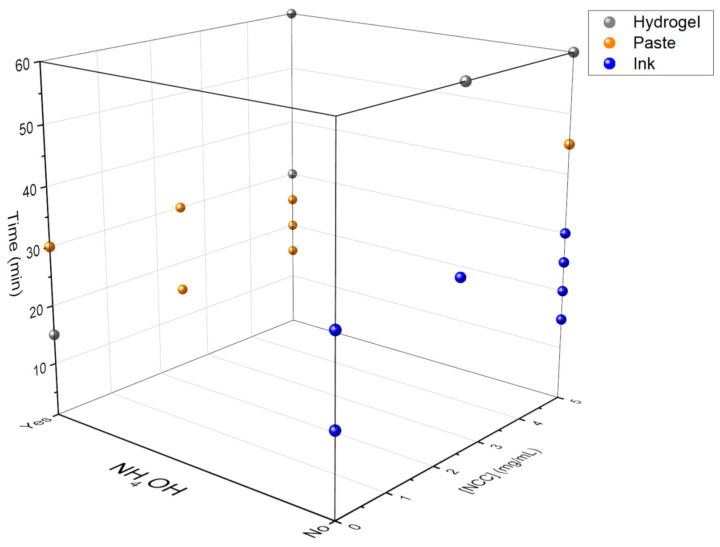
Three-dimensional scatter plot representing the conditions to obtain inks (blue), viscous pastes (orange) and self-standing hydrogels (gray). The effect of adding aqueous ammonia is herein represented, at different amounts of CNTs.

**Figure 3:**
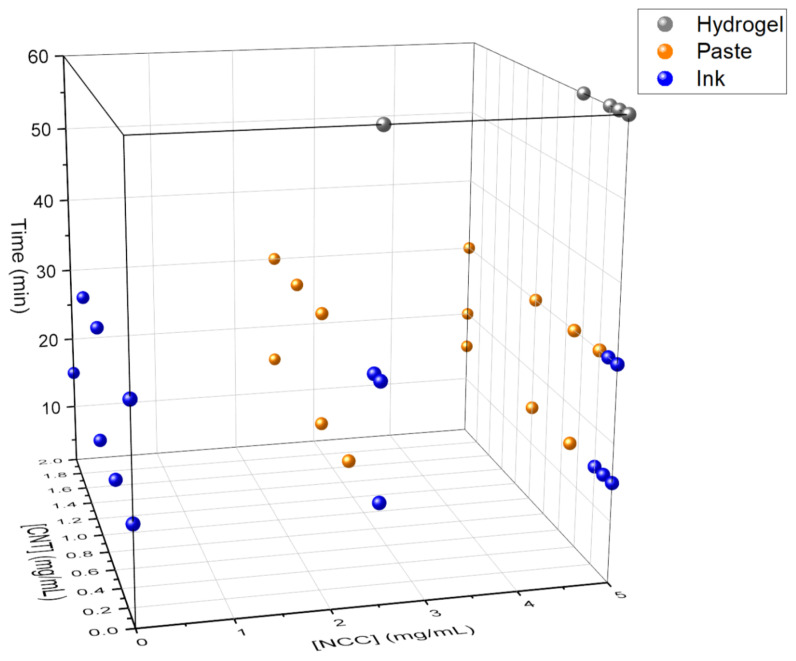
Three-dimensional scatter plot representing the conditions to obtain liquid inks (blue), viscous pastes (orange) and self-standing hydrogels (gray). The effect of CNTs is herein represented. All samples here had basic pH by adding aqueous ammonia.

As stated earlier, different experiments were carried out at different concentrations of NCC and CNTs, whereas the GO concentration remained fixed. Different hydrothermal treatment times were applied (always at 180 °C), and pH was also varied by adding NH_4_OH or not to the medium. The systematic combination of such variables provided a roadmap for this ternary system (CNTs, GO and NCC type I) in water upon hydrothermal treatment in an autoclave. The two main possibilities pursued in the present work were liquid inks and viscous pastes, but it is worth recalling that the hydrothermal treatment of aqueous GO suspensions can also provide self-standing hydrogels, even for short times (generally beyond 30–45 min at 180 °C), based on the crosslinking of the GO sheets [28]. The viscosity of the medium increases progressively with the treatment time, also aided by the presence of basic pH. Thus, by controlling the thermal treatment time, homogeneous liquid dispersions, pastes or hydrogels can be obtained (Figure 2 and Figure 3).

As observed in Figure 2 and Figure 3, hydrogels are always obtained for hydrothermal treatment times longer than 30 min, regardless of the other parameters. This suggests that treatment time is the dominant variable. This is consistent with our previous studies on the hydrothermal treatment of GO suspensions, with or without basic medium, in the absence of any other additive [28]. However, at 30 min of treatment time, the determining variable is the basicity of the medium. Samples containing NH_4_OH result in pastes or hydrogels, and those without NH_4_OH result in inks or pastes depending on the presence of CNTs. In fact, the joint presence of CNTs and NCC while adding NH_4_OH seems to be responsible for the rise in viscosity, leading to pastes.

For the experiments carried out for 15 min, the importance of the pH is observed again. Samples containing NH_4_OH give rise to pastes or hydrogels, and those without NH_4_OH give rise to inks. Again, the presence of NCC is decisive, since (at a given time of hydrothermal treatment with NH_4_OH) it leads to a paste, but in the absence of NCC the resulting outcome is a hydrogel. It can be reasonably postulated that the interactions between NCC and GO during hydrothermal treatment partly prevent the self-crosslinking of the GO and lead to viscous pastes instead of fully crosslinked hydrogels. Finally, it was observed that the concentration of NCC in the system was not a critical variable for the kind of aqueous formulations obtained. Therefore, in subsequent tests, this variable was set at 5 mg/mL.

In order to unravel the effect of crystalline polymorphism of NCC in the hydrothermal process, a series of experiments was also carried out with type II NCC. In this case, all experiments were performed for 15 min (since in the abovementioned results, there were no significant differences between 15 and 30 min). When NCC type II is incorporated into the system instead of type I, surprisingly, all formulations obtained in each experiment are low-viscosity inks (Table 1). This may be related to the aforesaid hindrance of GO crosslinking, as type II NCC could experience stronger interactions with the functional groups on the surface of GO, impeding its aggregation and thus leading to low-viscosity inks. This effect is stronger than that observed for type I NCC, which is consistent with the higher content of ester sulfate groups in type II NCC [22], responsible for its negative surface charge. As in the case for type I NCC, above 30 min of treatment time, in the presence of NH_4_OH, hydrogels are always obtained with type II NCC.

### 3.2. Morphology of Films Derived from Inks and Pastes

**Figure 4:**
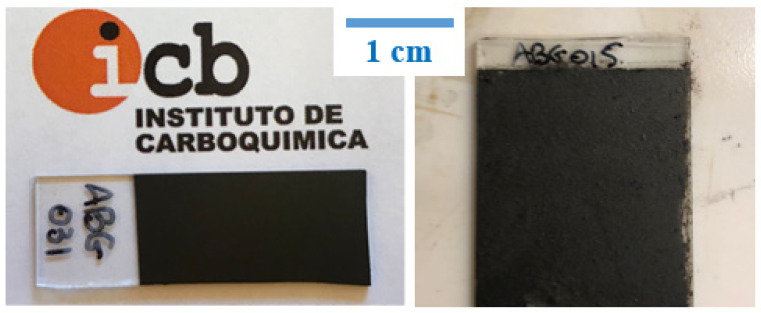
Photographs of films made from low-viscosity inks (**left**) and high-viscosity pastes (**right**) on glass substrates.

**Figure 5:**
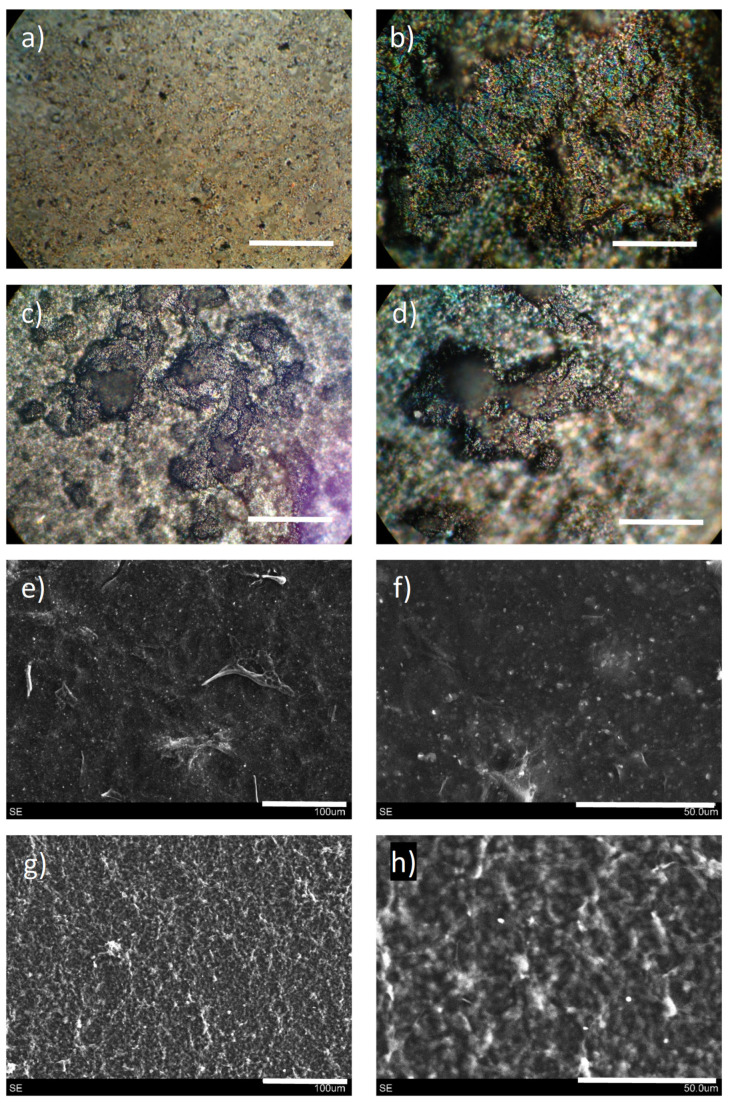
Optical photographs (**a**–**d**) and SEM images (**e**–**h**) of films obtained with low-viscosity inks (**a**,**b**,**e**,**f**) and high-viscosity pastes (**c**,**d**,**g**,**h**). Scale bars (in white) = 1 mm (**a**,**b**), 400 µm (**c**,**d**), 100 µm (**e**,**g**) and 50 µm (**f**,**h**). Each image (either optical or from SEM) corresponds to a random point of each sample, none is the direct magnification of another.

Low-viscosity inks and high-viscosity pastes require the use of different deposition methods to attain conductive films over glass substrates. Inks were deposited by means of spray coating, while pastes were processed using the rod-coating method. The general appearance of such films is depicted in Figure 4. Films made from inks had a very compact aspect, because spray coating allowed for a tight coverage. Viscous pastes could not be deposited by spraying, with the rod coating method being the one showing the highest effectiveness, leading to films with an apparently more porous aspect. Deeper insights into the surface morphology of these films were obtained by both optical and scanning electron microscopies (Figure 5).

As confirmed by microscopy images, films derived from liquid inks and viscous pastes have a very distinct morphology both at the millimeter and the micrometer scale. According to the millimeter scale, films derived from inks exhibit no major irregularities, and a negligible presence of pores. In contrast, those coming from rod-coated pastes show the presence of large pores, together with more irregularities. These pores may be important for subsequent processing of such films, such as liquid-phase infiltration or interfacing with other species. As for the film surface microstructure, unraveled by SEM, the observation from optical microscopy is corroborated. Films derived from inks present higher homogeneity and compaction, while those coming from pastes are more topographically irregular. In essence, both kinds of films display a very unique topography.

**Figure 6:**
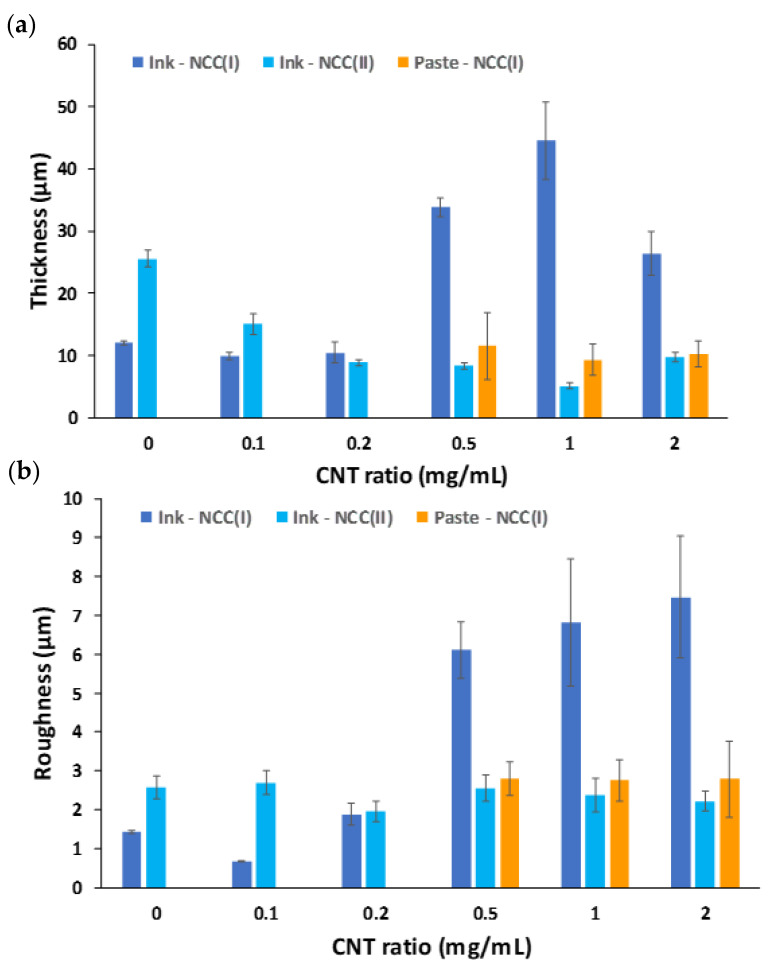
Film thicknesses (**a**) and roughnesses (**b**) corresponding to samples coming from sprayed inks or rod-coated pastes.

**Figure 7:**
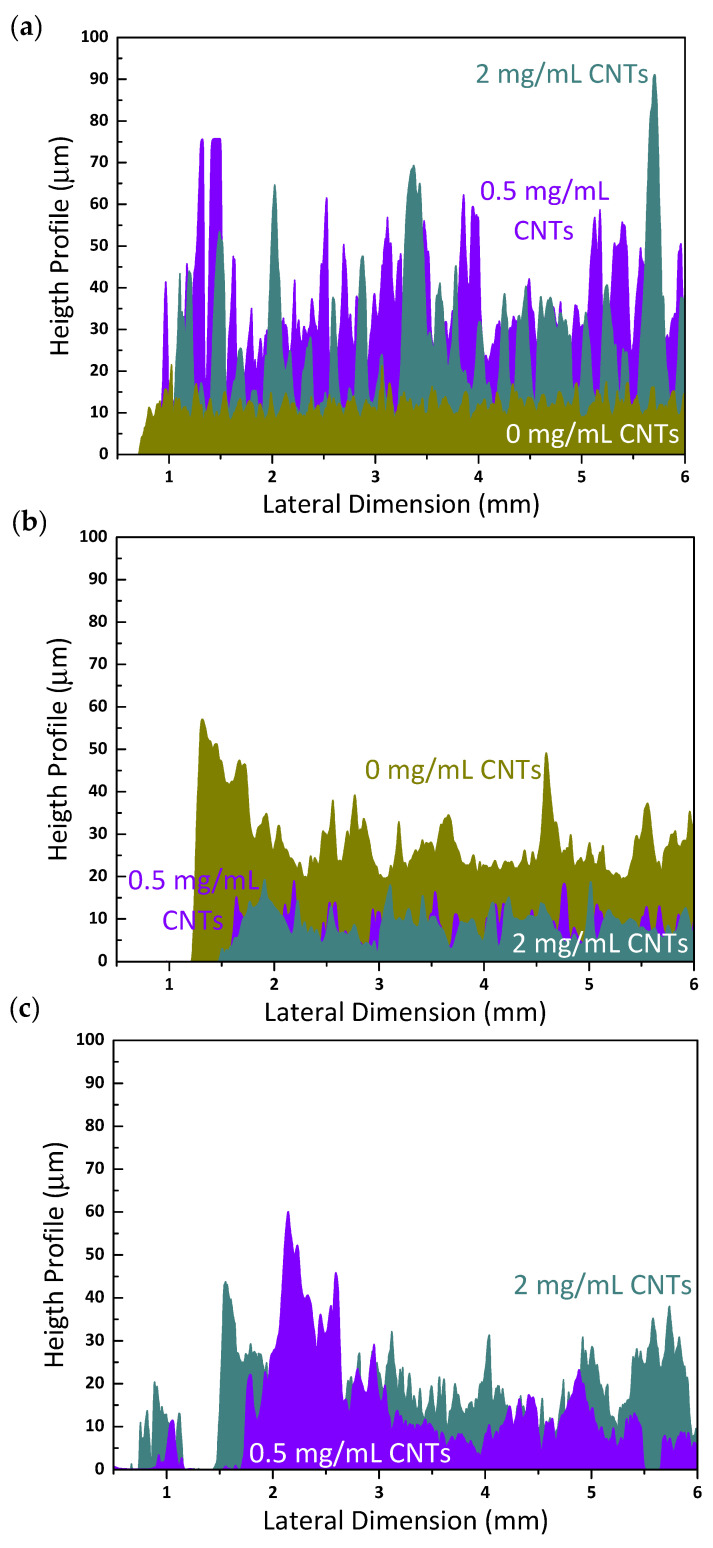
Topographic profiles across 6 mm distance for films made from inks with type I NCC (**a**), from inks with type II NCC (**b**) and from pastes with type I NCC (**c**), at the indicated CNT ratio in the initial formulation. All samples came from formulations with 2 mg/mL GO and 5 mg/mL NCC.

Additional results were obtained through the profilometer, by which thickness and roughness were quantified. The mean values for the films studied in the present work are shown in Figure 6. Inks fabricated with type I NCC are able to generate films of a low thickness (~10 µm on average) up to a certain CNT content. From 0.5 mg/mL CNTs and up, the sprayed films in identical conditions drastically increase in thickness, reaching values in the range of ~30–45 µm. Conversely, inks containing type II NCC present the opposite trend; at a null content of CNTs, the sprayed films display an average thickness of ~25 µm and progressively decrease with increasing content of CNTs. Films made from pastes showed a steady value regardless of the CNT content, with an average of ~10–15 µm thickness. Film roughness shows an identical trend as thickness for inks with type I NCC; low roughness (~1–2 µm) until reaching 0.5 mg/mL CNTs, after which this value becomes 3- or 4-fold larger. Inks from type II NCC and pastes lead to films of comparable roughness, in the range of ~2–3 µm. All of these data may be related to the different interaction that NCC has with carbon nanostructures. In a previous work [24], we reported that type I NCC hybrids with CNTs presented a discrete distribution of cellulose nanocrystals adsorbed on the sidewalls, while type II NCC led to a heavily wrapped nanohybrid. In addition, type I cellulose nanocrystals are much longer and needle-like whereas the type II ones are much shorter and slightly thicker [22]. The kind of interaction among nanostructures in the reaction mixture during the hydrothermal treatment could determine their packing upon film deposition. Spraying liquid inks with type I NCC may provide a less efficient packing of nanostructures when the CNT content is high enough, causing the thickness and roughness to increase. In contrast, type II NCC, could have led to efficient packing regardless of the CNT content, given its very distinct structure. As for pastes, the explanation could lie in the nature of the deposition technique, as rod-coating of viscous formulations may not provide a tight packing nor attain highly thick or rough surfaces. Additionally, the rod-coating method also exhibited a visible thickening of the film edges. These observations can be better understood by observing some representative profiles of each case (Figure 7). Films coming from both inks show a more regular aspect with periodic spikes in the presence of CNTs, which determine the higher or lower mean roughness, while pastes present a more irregular profile, with some visible pores in the millimeter scale, as observed by optical microscopy. The profilometer also served as a means to perform a scratching test on selected film samples (see Supplementary Materials, Video S1). The conclusions drawn from these tests is that the films have a high adhesion and mechanical resistance, since they are not damaged at all when scratched with high local pressures (3.75 MPa).

A final surface characterization of these films was conducted through Raman spectroscopy (Supplementary Materials, Figure S4). In the observed spectra, it is visible how the D-band (~1349 cm^−1^) and the G-band (~1590 cm^−1^) correspond to both multi-walled CNTs and reduced GO indistinguishably, but are different from the starting GO due to the better definition of the band at 2700 cm^−1^ (2D) in the films, and the rougher profile of GO. The D/G intensity ratio is lower than 1 for GO and films without CNTs, while in the presence of the latter, this D/G ratio may reach values of ≥1.

### 3.3. Characterization of Electrical Properties

**Figure 8:**
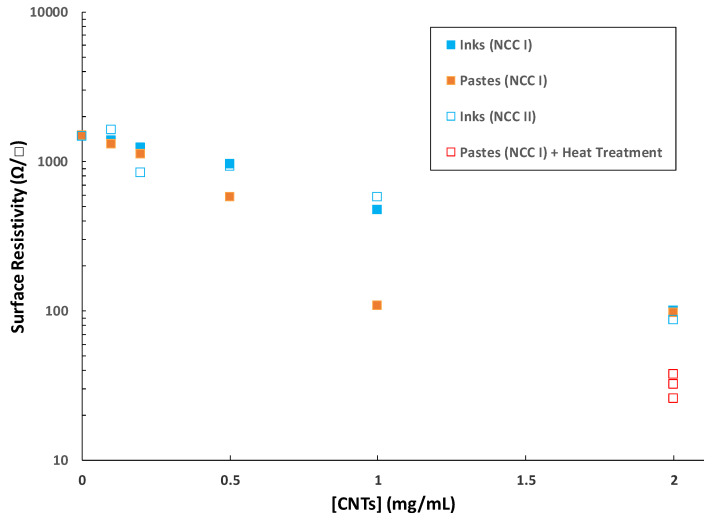
Surface resistivity measurements for different films prepared from inks and pastes. The concentration of CNTs refers to the one in the liquid formulation before deposition. All samples on this graph came from formulations with 2 mg/mL GO and 5 mg/mL NCC.

The four-probe electrical measurements performed on the prepared films allowed us to obtain their surface resistivity (Figure 8). It is observed that the surface resistivity values are lower as the concentration of CNTs in the liquid formulation (ink or paste) increases. Indeed, if the presence of GO is the basis for the hydrothermal aggregation, and the incorporation of NCC exerts control over the viscosity of the medium, the control over the conductive behavior of the derived films can be ascribed to CNTs. Those films without CNTs present very similar resistivity values amongst them (~1.5·10^3^ Ω/□), regardless of whether they come from more or less viscous formulations before deposition, most likely owing to the lone effect of hydrothermally reduced GO. In fact, NCC does not generate any kind of char in the working conditions (Figure S3, Supplementary Materials). Regarding the inks, there are no significant differences in surface resistivity between those films coming from type I or II NCC. Both kinds of inks provide films with surface resistivities in the range of ~10^3^–10^2^ Ω/□ up to 1 mg/mL CNTs, reaching further values below 100 Ω/□ at 2 mg/mL CNTs. All these values decrease almost linearly on a logarithmic scale, typical for a behavior beyond the percolation threshold in the studied conditions. Films made from pastes exhibit a steeper decrease in the surface resistivity with an increasing amount of CNTs, attaining ~100 Ω/□ at 1 mg/mL CNTs. This seems to be a ‘plateau’ value as resistivity does not change at 2 mg/mL CNTs. It is worth pointing out that these films could become perfect candidates for metal-free electrode components or current collectors in liquid-phase processed electric/optoelectronic layered film device structures. In order to account for the variation trends in film thickness with increasing CNT content (Figure 6), the bulk conductivity of the samples could also be calculated (see Figure S5, Supplementary Materials). These results also show a generally direct proportionality between both parameters.

### 3.4. Post-Synthesis Processing Versatility

Up to this point, we have presented a green approach to water-based inks with carbon nanostructures. This has led to formulations of varied viscosity able to be deposited by different means, leading to conductive films with good surface resistivity, unique surface morphologies and a potential feasibility to be applied as electrodes. Nonetheless, the properties of such films may be still improved, in terms of electrical conductivity and tolerance to organics, upon heat treatment. As a proof of concept, we chose a candidate film presenting one of the lowest values of surface resistivity (97.4 Ω/□, coming from a rod-coated paste with 2 mg/mL GO, 5 mg/mL NCC and 2 mg/mL CNTs), and subjected it to heat treatment and organic solvent exposure.

**Figure 9:**
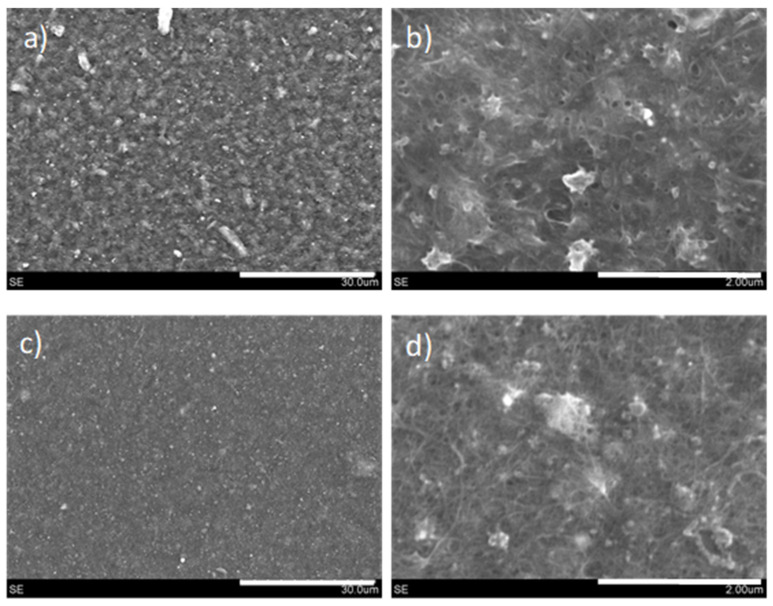
SEM images from films obtained with high-viscosity pastes, before (**a**,**b**) and after (**c**,**d**) heat treatment at 400 °C. Scale bars (in white) = 30 µm (**a**,**c**) and 2 µm (**b**,**d**).

In one experiment, the selected film was inserted into an oven and heated at 400 °C for 4h under air atmosphere, and the outcome was a film with intact integrity and lower surface resistivity (Figure 8). The production of three different replicas provided values in the range of ~25–35 Ω/□, being of special interest for the construction of conductive carbon layers in layered film optoelectronic devices [29]. The morphology of the films before and after heat treatment was assessed again by SEM (Figure 9). It becomes clear that the effect of heat treatment is to smoothen the surface topography, probably together with the burning of NCC. In the heat-treated films, the presence of CNTs is better discerned, very probably entailing an improved contact between CNTs and reduced GO, thus leading to lower interparticle contact resistance. These results are very encouraging for the replacement of commercial carbon pastes with many toxic additives and binders, as these pastes have been reported to peel off and deform when heated beyond 250–300 °C [30].

An additional advantage is the stability over time of the conductive properties of the films. Along the course of this research, we corroborated the stability of the conductive properties of our films in a time frame of many hours or a few days, meaning that the measurement of the surface resistivity of a freshly prepared film is generally coincident with the measurement after a short–medium time frame. Additionally, these observations are also valid for long-term periods (months). All of this shows that the preservation of the conductive properties of these films is possible across lengthy time periods with regular shelf storage.

In another experiment, the films were subjected to a treatment with typical organic solvents in the LPP of carbon nanostructures (NMP, DMSO), in order to study the tolerance of the films to such solvents. For this, the films were heated at 60 °C for 5 min. Then, several droplets of one of these organic solvents in a volume of 45 μL were randomly scattered across different areas of the films, in order to evaluate their effect towards its integrity. The film was maintained at 60 °C for 10–15 min to ensure infiltration through the pores and placed in the oven at 60 °C for 4 h. The film perfectly resisted this treatment, as no evident damage was observed by eye nor the optical microscope, and no peeling off occurred either. In addition, its surface resistivity changed from 97.4 to 94.4 Ω/□, which could be considered negligible and within experimental error.

In essence, these tests reassert the robustness and versatility of our conductive films, as they can endure (and be improved in terms of surface resistivity by) thermal treatments at high temperatures and long durations, as well as withstand the exposure to high boiling point organic solvents in aggressive conditions without any morphological harm nor damage to the electrical properties. This latter fact is of special relevance for their future performance as electrode components, since the whole integrity and surface resistivity of the films are retained after aggressive treatments, and depending on the application in mind, the resistance of the films towards certain critical solvents could be an important advantage.

### 3.5. Properties of Hydrogel-Derived Aerogels

**Figure 10:**
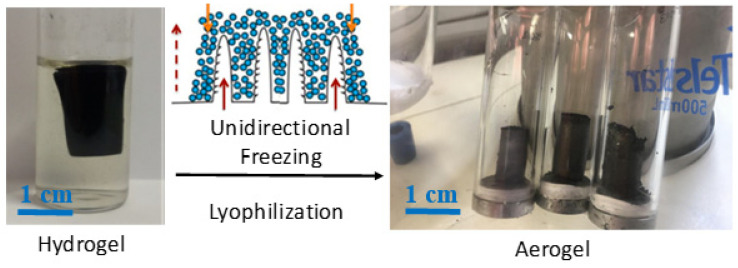
Real images of a hydrogel derived from hydrothermal treatment in water (**left**) and preparation scheme of aerogels by unidirectional freezing followed by lyophilization (**right**).

Some of the working conditions led to porous and light graphene-based aerogels [26,28]. We have taken advantage of the possibilities granted by unidirectional freezing, followed by lyophilization, which are able to create an anisotropic internal microstructure composed of parallel channels, with critical applications in energy and environmental remediation [28,31,32]. In the present case, hydrogels were prepared under specific conditions and subjected to hydrothermal treatment with the ternary GO/NCC/CNT system in water (see Section 3.1 and Figure 10), and when subjected to unidirectional freezing prior to lyophilization, they also presented an anisotropic porous microstructure.

**Figure 11:**
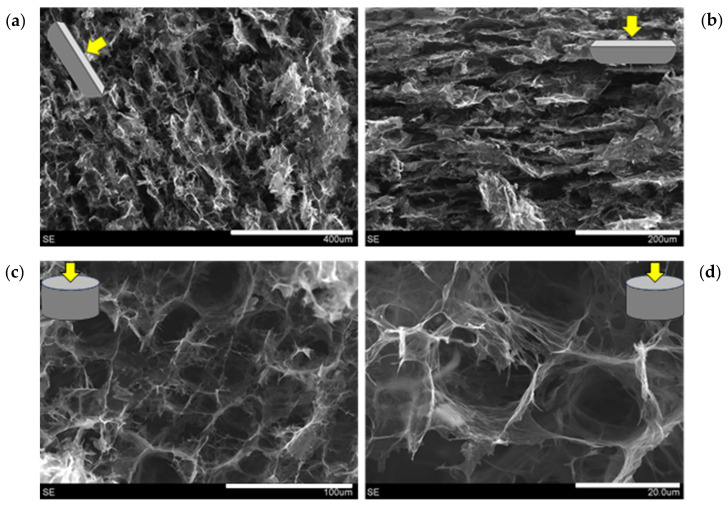
SEM images of aerogels derived from the unidirectional freezing followed by lyophilization of hydrothermally prepared hydrogels. Longitudinal (**a**,**b**) and transversal (**c**,**d**) cuts. Scale bars (in white): 400 µm (**a**), 200 µm (**b**), 100 µm (**c**) and 20 µm (**d**).

As inferred from the SEM images (Figure 11), aerogels resulting from the unidirectional freeze-drying of hydrogels presented an anisotropic microstructure, with continuous straight pores parallel to the aerogel’s longitudinal axis. This demonstrates that not only can such a microstructure be obtained from the reduction of GO in hydrothermal conditions [28], but also in the presence of NCC and CNTs. According to measurements of weight and dimensions, we elucidated that these aerogels presented an average density of 0.028 ± 0.006 g/cm^3^, and axial resistivities (measured in a two-probe configuration) in the range of 10–100 Ω·m, for CNT concentrations in the range of 0.1 to 0.5 mg/mL. Aerogels obtained in similar conditions but without CNTs or NCC presented lower densities (~0.005 g/cm^3^), but higher electrical resistivity (~1000 Ω·m). Parallel to the case of inks and pastes, the presence of CNTs governs the electrical properties of freeze-dried hydrogels, but in this case, NCC has a significant influence. When only GO and NCC are present in the hydrothermal medium, the density rises by one order of magnitude (~0.036 g/cm^3^) with respect to GO-only aerogels, but the axial resistivity is lowered by one order of magnitude. Therefore, all aerogels with GO, CNTs and NCC display electrical resistivities from ~10^2^ down to ~10^0^ in the studied range (from 0.1 to 0.5 mg/mL), revealing the importance of NCC in the process, which seems to play a role in the GO reduction during the hydrothermal treatment. NCC seems to boost the GO hydrothermal reduction and the number of interparticle contacts, hence favoring the decrease in electrical resistance at the expense of aerogel density. In summary, it is possible to produce light and porous aerogels with a GO/CNT/NCC ternary system, with an anisotropic microstructure and fairly low electrical resistivity in the absence of any post-treatment, comparable to the ones with only GO [32].

## 4. Conclusions

Nanocrystalline cellulose has been demonstrated to be a green and sustainable means to generate conductive water-based ink formulations of tailored viscosity, with carbon nanotubes and graphene oxide, as well as three-dimensional structures. Control of the processing parameters allows the preparation of inks, pastes or hydrogels using the same approach. The liquid inks and pastes have been used to fabricate films exhibiting diverse surface morphologies (depending on the deposition method and composition), with low resistivity values (<100 Ω/□). These conductive films also exhibit great robustness, being able to avoid disintegration upon aggressive treatments with organic solvents, as well as being electrically improvable by high-temperature treatments. Our studies show that formulations of this kind have great potential as metal-free electrodes in liquid-phase processed layered films, devices and structures. The green processes herein described, in addition to the capability of easily tuning the viscosity of liquid formulations, can be a great starting point for industrial development.

## Figures and Tables

**Table 1:** The outcome of the hydrothermal treatment and composition of the GO/CNT/NCC (type II) ternary system in water at 180 °C for 15 min.

Formulation Obtained	GO (mg/mL)	CNTs (mg/mL)	NCC (mg/mL)	V_H2O_ (mL)	V_NH4OH_ (mL)
Ink	2	0.0	5	5	0
Ink	2	0.0	5	4.8	0.2
Ink	2	0.1	5	5	0
Ink	2	0.1	5	4.8	0.2
Ink	2	0.2	5	5	0
Ink	2	0.2	5	4.8	0.2
Ink	2	0.5	5	5	0
Ink	2	0.5	5	4.8	0.2
Ink	2	1	5	5	0
Ink	2	1	5	4.8	0.2
Ink	2	2	5	5	0
Ink	2	2	5	4.8	0.2

## Data Availability

Data sharing is not applicable to this article.

## Supplementary Materials

The following are available online at https://www.mdpi.com/article/10.3390/nano11061435/s1, Figure S1: Thermogravimetric analysis in N_2_ atmosphere, Figure S2: TEM images of mildly oxidized MWCNTs, Figure S3: Photograph of NCC aqueous colloids subjected to hydrothermal treatment. Figure S4. Raman spectra (measured with a 532nm laser, Horiba Jobin Yvon equipment) for different selected conductive films. Each spectrum is the average of at least 5 random points across the film surface. Figure S5. Electrical conductivity values for different films coming from inks or pastes with different carbon nanotube contents. Videos S1: Recording of the stylus-based scratching tests, by applying a constant pressure of 3.75 MPa with the diamond tip (in the center of the crosshead). The distance traveled was 3 mm in each case. After scratching the films, no grooves or trails were observed, and the final profile seen corresponds to the neat sample topography. This means that these films are not damaged at all when scratched at high local pressures.
