# Supplementary material for: Waterborne Graphene- and Nanocellulose-Based Inks for Functional Conductive Films and 3D Structures
Source: Nanomaterials (Basel). 2021 May 29;11(6):1435. doi: 10.3390/nano11061435 (PMC8227753; doi:10.3390/nano11061435)
Supplement: Supplementary file 1 [file nanomaterials-11-01435-s001.zip › nanomaterials-1228592-SI.pdf]

SUPPLEMENTARY MATERIALS

Waterborne Graphene- and Nanocellulose-Based Inks for Functional Conductive Films and 3D Structures

Jose M. González-Domínguez [1,*], Alejandro Baigorri [1], Miguel Á. Álvarez-Sánchez [1], Eduardo Colom [1], Belén Villacampa [2], Alejandro Ansón-Casaos [1], Enrique García-Bordejé [1], Ana M. Benito [1] and Wolfgang K. Maser [1]

[1] Instituto de Carboquímica ICB-CSIC, C/Miguel Luesma Castán 4, 50018 Zaragoza, Spain; baigorri1994@gmail.com (A.B.); maalvarez@icb.csic.es (M. Á. Á.-S.); ecolom@icb.csic.es (E.C.); alanson@icb.csic.es (A.A.-C.); jegarcia@icb.csic.es (E.G.-B.); abenito@icb.csic.es (A.M.B.); wmaser@icb.csic.es (W.K.M.)

[2] Department of Condensed Matter Physics, ICMA-CSIC, University of Zaragoza, 50009 Zaragoza, Spain; bvillaca@unizar.es

* Correspondence: jmgonzalez@icb.csic.es

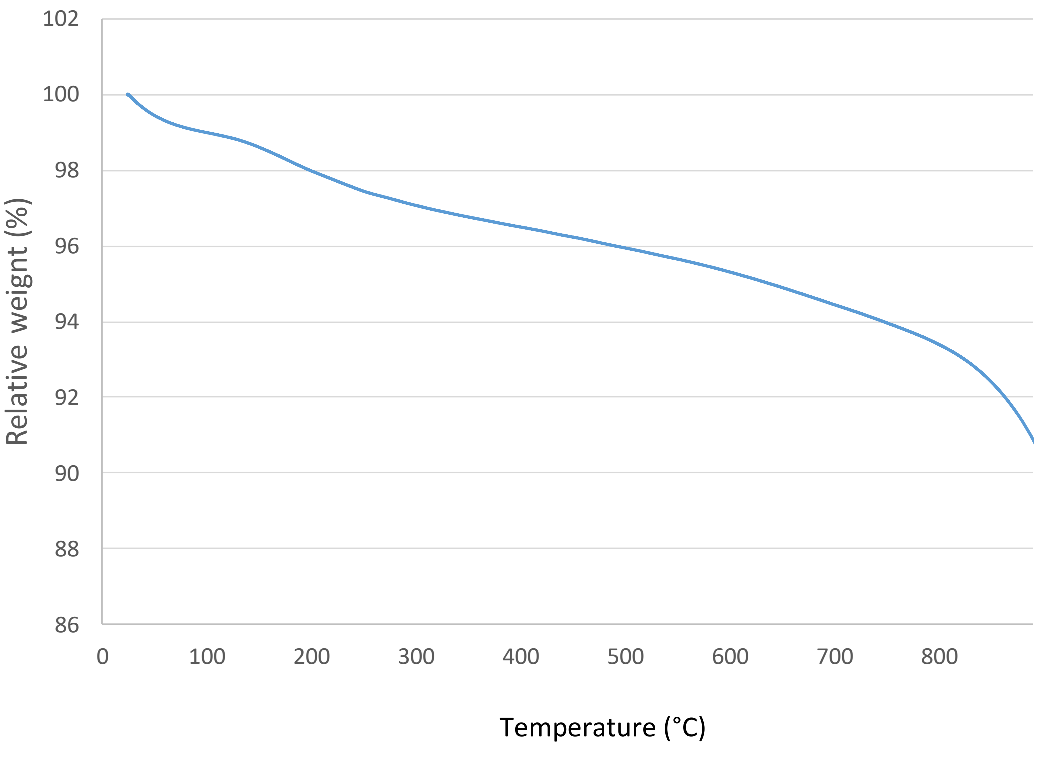

Figure S1. Thermogravimetric analysis in N_2 atmosphere (10 °C/min), revealing that mildly oxidized MWCNTs presented about 6 wt% oxygen functional groups in their structure.

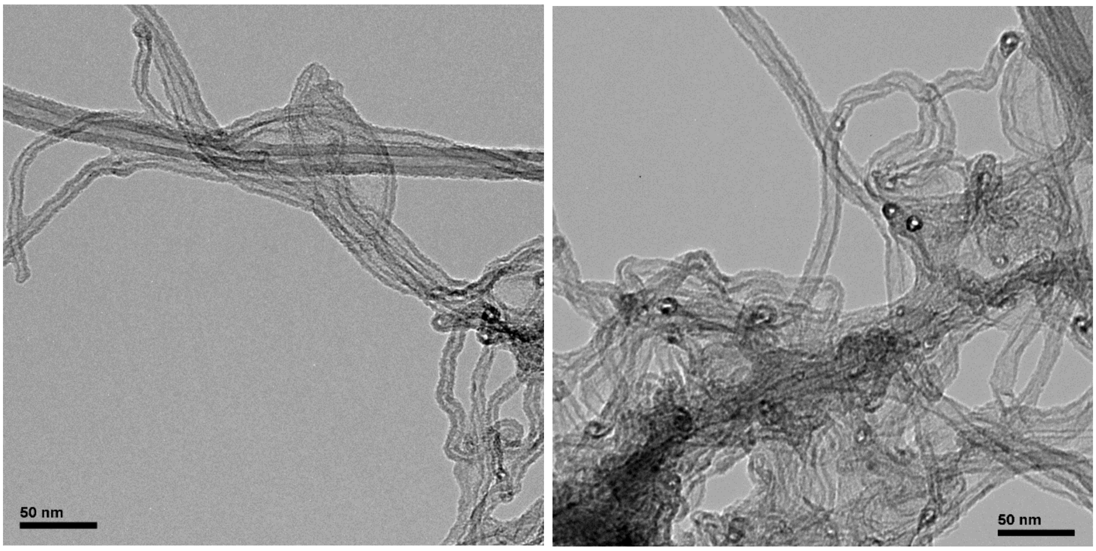

Figure S2. TEM images of mildly oxidized MWCNTs, in which the white dots are holes after leaching out the metallic impurities.

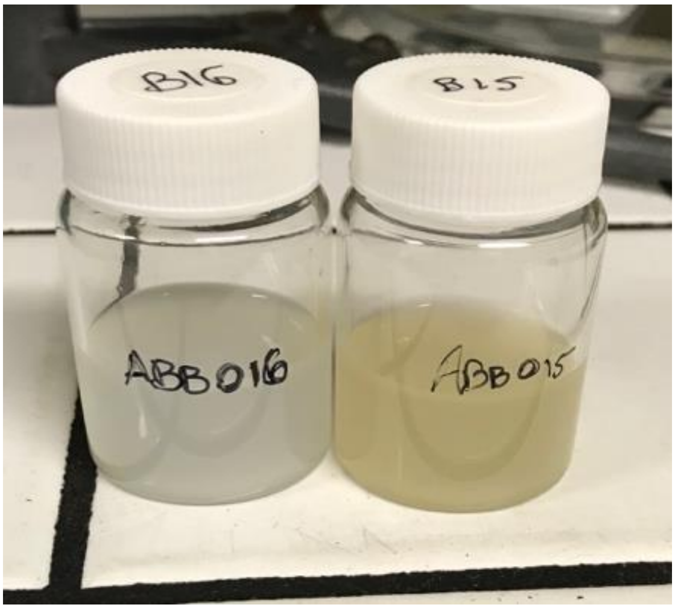

Figure S3. Photograph of NCC aqueous colloids subjected to hydrothermal treatment (180 °C in autoclave, with addition of NH_4OH) in the absence of any carbon nanomaterial. Left: 15 min, Right: 30 min. Only a slight caramelization is observed from 30 min on, but no burning or charring of the sample was noticed.

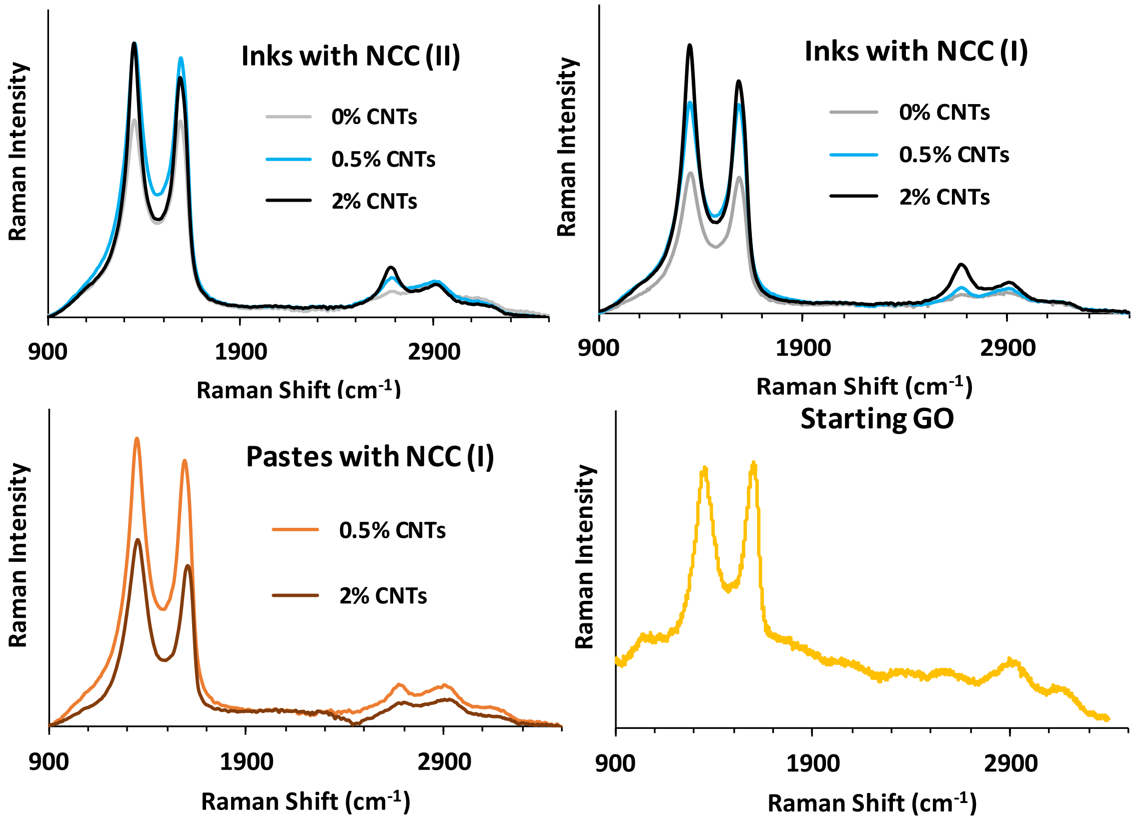

Figure S4. Raman spectra (measured with a 532nm laser, Horiba Jobin Yvon equipment) for different selected conductive films. Each spectrum is the average of at least 5 random points across the film surface.

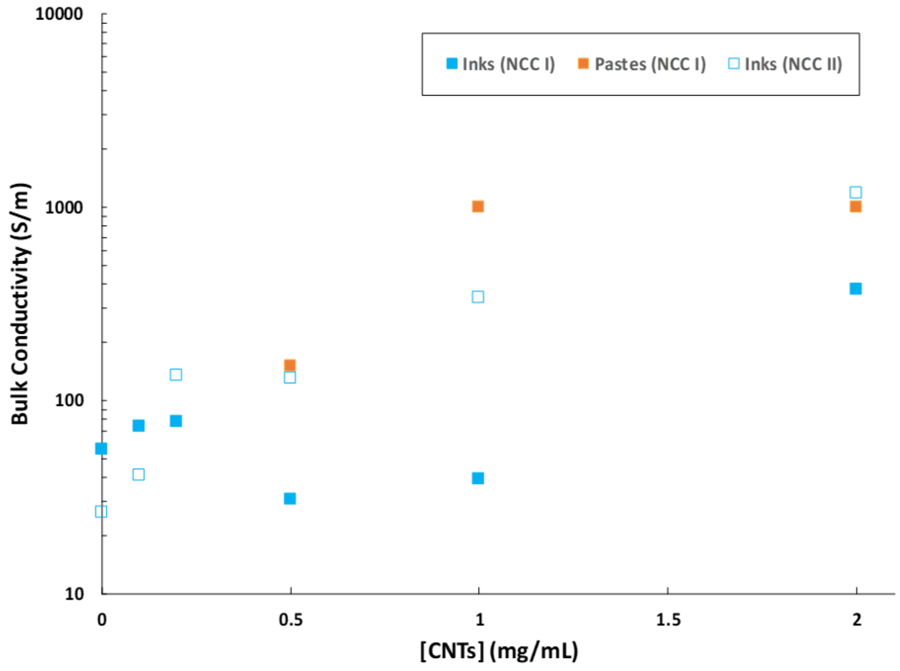

Figure S5. Electrical conductivity values for different films coming from inks or pastes with different carbon nanotube contents.

Videos S1

1. Ink with NCC –I (2% CNTs)

2. Paste with Ink with NCC –II (2% CNTs)

3. Ink with NCC –II (2% CNTs)

Recording of the stylus-based scratching tests, by applying a constant pressure of 3.75 MPa with the diamond tip (in the center of the crosshead). The distance traveled was 3 mm in each case. After scratching the films, no grooves or trails were observed, and the final profile seen corresponds to the neat sample topography. This means that these films are not damaged at all when scratched at high local pressures.
